# The CD14 (−159 C/T) SNP is associated with sCD14 levels and allergic asthma, but not with CD14 expression on monocytes

**DOI:** 10.1038/s41598-018-20483-1

**Published:** 2018-03-07

**Authors:** J. J. Nieto-Fontarigo, F. J. Salgado, M. E. San-José, M. J. Cruz, A. Casas-Fernández, M. J. Gómez-Conde, L. Valdés-Cuadrado, M. Á. García-González, P. Arias, M. Nogueira, F. J. González-Barcala

**Affiliations:** 1 Department of Biochemistry and Molecular Biology, Faculty of Biology-Biological Research Centre (CIBUS), Universidade de Santiago de Compostela, Santiago de Compostela, Spain; 2 Clinical Analysis Service, University Hospital of Santiago de Compostela (CHUS), Santiago de Compostela, Spain; 3 Department of Respiratory Medicine-Hospital Vall d’Hebron, Universitat Autònoma de Barcelona, Barcelona, Spain; 4 Spanish Biomedical Research Networking Centre-CIBERES, Santiago de Compostela, Spain; 5 Department of Medicine, University of Santiago de Compostela, Santiago de Compostela, Spain; 6 Health Research Institute of Santiago de Compostela (IDIS), Santiago de Compostela, Spain; 7 Department of Respiratory Medicine, University Hospital of Santiago de Compostela (CHUS), Santiago de Compostela, Spain

## Abstract

LPS-ligation to CD14/TLR-4 on monocytes/macrophages triggers the production of IL-12-family cytokines. IL12/18 promote TH_1_-differentiation, counteracting the TH_2_-driven asthma. Therefore, CD14 modulation could alter the TH_2_-differentiation and should be taken into account when studying asthma. To analyse the alteration in CD14 levels and its association with *CD14* (−159 C/T) SNP (rs2569190) in Caucasian adults with stable allergic asthma, we performed a cross-sectional study (277 healthy subjects vs. 277 patients) where clinical parameters, CD14 values and the *CD14* (−159 C/T) SNP were studied. Apart from typical biomarkers, we found an increment of neuron-specific enolase (NSE) in allergic asthma, probably linked to monocyte activity. Indeed, we evidenced increased monocyte numbers, but lower CD14 expression and normalised sCD14 values in patients. Moreover, we noticed an association of the T allele (*P* = 0.0162) and TT genotype (*P* = 0.0196) of the *CD14* SNP with a decreased risk of allergic asthma and augmented sCD14 levels. In conclusion, monocyte CD14 expression and normalized sCD14 values were reduced in stable state asthmatics, and this could be related to the presence of an expanded CD14^low^ monocyte subset. This study also demonstrates that the *CD14* (−159 C/T) polymorphism is a risk factor for moderate-severe allergic asthma in adult Caucasians.

## Introduction

During asthma attacks allergens trigger lung epithelial cells to release cytokines (e.g., Thymic stromal lymphopoietin (TSLP), Interleukin (IL)-33, IL-25) that activate innate leukocytes and drive the differentiation of allergen-specific T helper (TH)_2_ lymphocytes^1^. Innate defences also rely on pattern recognition receptors, such as toll-like receptors (TLRs), capable of detecting pathogen-associated molecular patterns (PAMPs). Lipopolysaccharide (LPS) is a PAMP that interacts with CD14 (monocytes, macrophages, and neutrophils)^2^, a receptor whose gene has been linked to asthma/allergy^3^. CD14 transfers LPS to the TLR4-MD2 complex, which induces the secretion of a set of cytokines (IL-12/IL-18) that act as an innate-adaptive bridge and favour the TH_1_ differentiation^4^. In addition, IL-12 also prevents the development of allergen-specific TH_2_ cells, which ameliorates airway inflammation in allergic asthma^4^. Therefore, a functional defect in CD14 could alter TH_2_ differentiation and IgE-mediated allergic diseases^5^.

Membrane-bound CD14 (mCD14) is released to the medium as soluble CD14 (sCD14)^2,6,7^, which either potentiates the response to LPS in macrophages by inducing the release of proinflammatory cytokines^8^, or has a protective role by transferring LPS to lipoproteins^9^. sCD14 is released from monocytes through a process enhanced by LPS, IL-6 and IL-1β^6^, but decreased by interferon-γ (IFN-γ) and IL-4^10^. sCD14 inversely correlates with IL-4^11^, but positively with the number of sputum eosinophils^12^. Moreover, plasma sCD14 is a biomarker of ongoing or acute immune responses^2,13^. Thus, children with asthma exacerbations display augmented sCD14 levels compared to the recovery phase^14,15^, whereas adult asthmatics have higher amounts in sputum than healthy subjects^12^. An increased sCD14 concentration has also been detected in bronchoalveolar lavage fluid (BALF) upon allergen exposure^16^. However, other works reported unaltered sCD14 levels, like for example in asthmatic children^7,15^.

Single-nucleotide polymorphisms (SNPs) also constitute modulatory agents for CD14 levels that could influence the risk factors for developing asthma or aggravate disease symptoms^17^. Baldini *et al*. found an association between *CD14* (−159 C/T) SNP (rs2569190) and sCD14 in atopic children, showing TT homozygotes higher levels than CT/CC genotypes^11^ in line with the enhanced *CD14* transcriptional activity in the T allele^5^. Since those landmark studies, different works have confirmed these results^18,19^, while the C allele has been associated with high IgE levels, atopy and asthma^15,17,20^. However, this association has not been consistently replicated^3,13,21–24^. These different results may be attributed to a number of factors, like the low sample size, asthma phenotype (atopic, non-atopic or mixed asthma), age of study population (children or adults), ethnicity (African, Caucasian or Asiatic population) or gene-environment interactions (e.g., endotoxin levels)^21,25^. Therefore, it seems necessary to undertake new works aimed to the simultaneous measurement of both membrane and soluble forms of CD14 as well as the *CD14* (−159 C/T) SNP in adult Caucasian subjects with allergic asthma and different degrees of severity (intermittent-mild and moderate-severe).

## Results

### Demographic and clinical characteristics of the study population

In this work a case-control study was performed, where adult patients with both intermittent-mild and moderate-severe allergic asthma were recruited. These patients were in a clinically stable state, had a well controlled disease, and the majority were non-smokers under treatment with inhaled corticosteroids (Table 1). FEV1 (%) and FEV1/FVC ratio (%) values are described in Table 1, showing decreased levels in MSAA (moderate-severe allergic asthmatics) compared to IMAA (intermittent-mild allergic asthmatics) (Table 1). Although 66.4% of AA came from rural areas, most of them had no animals or just dogs/cats; only 11% were taking care of farm animals.

**Table 1:** Characteristics of the study population.

	**AA**			**HC**
	**ALL**	**IMAA**	**MSAA**	
N	277	108	169	277
Age^a^	32 (25–42)	31 (22–38)	33 (26–46)	46 (31–60)
Sex (M/F)	119/158	42/66	77/92	105/172
Smokers (%)	21.3	15.7	24.8	0
Control				
Good	219	98	121	—
Partial	36	8	28	—
Bad	22	2	20	—
Treatment:				
No	66	66	0	—
Inhaled Corticosteroids	196	34	162	—
Oral Corticosteroids	15	0	15	—
Antileukotrienes	63	6	57	—
Omalizumab	4	0	4	—
FEV1 (%)	97.2 (83.7–108.0)	105.0 (95.3–115.7)^#^	93 (74.5–102.2)^#^	—
FEV1/FVC (%)	78.0 (70.7–85.4)	82.9 (75.9–88.4)^#^	74.7 (67.6–81.9)^#^	—
Neutrophils (10^3^ cells/μL)^a^	3.67 (2.77–4.50)	3.53 (2.99–4.27)	3.71 (2.90–4.96)	3.54 (2.77–4.50)
Lymphocytes (10^3^ cells/μL)^a^	2.22 (1.85–2.71)*	2.20 (1.86–2.79)^&^	2.23 (1.80–2.71)^&^	2.02 (1.62–2.45)
Monocytes (10^3^ cells/μL)^a^	0.47 (0.38–0.59)*	0.47 (0.39–0.56)^&^	0.46 (0.37–0.62)^&^	0.35 (0.28–0.43)
Eosinophils (10^3^ cells/μL)^a^	0.33 (0.19–0.51)*	0.29 (0.20–0.51)^&^	0.34 (0.18–0.51)^&^	0.16 (0.11–0.23)
Basophils ((10^3^ cells/μL)^a^	0.04 (0.03–0.05)	0.04 (0.03–0.05)	0.04 (0.02–0.06)	0.04 (0.03–0.06)
IgE (IU/mL)^a^	272 (116–533)*	270 (111–485)^&^	276 (122–668)^&^	34 (11–102)

AA, allergic asthmatics; HC, healthy controls; IMAA, intermittent-mild allergic asthmatics; MSAA, moderate-severe allergic asthmatics.

^a^Median value (IQR1–3); *AA vs HC: Mann-Witney U Statistic, *P* < 0.001; ^#^IMAA vs MSAA: Mann-Witney U Statistic, *P* < 0.001; ^&^IMAA vs MSAA vs HC: Kruskal-Wallis One Way Analysis of Variance on Ranks (P < 0.001), IMAA/MSAA vs HC P < 0.05 Dunn’s Method.

**Figure 1:**
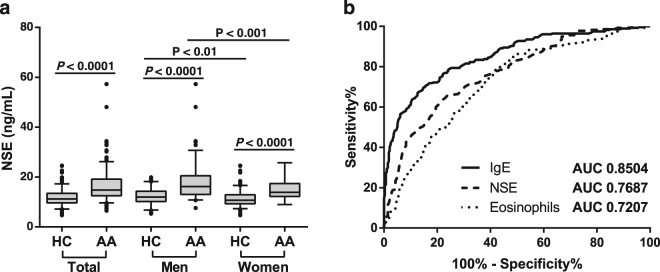
Analysis of NSE values in serum from AA and HC. (**a**) NSE levels in allergic asthmatics (AA) and healthy controls (HC) segregated by gender. Numbers on the graphs represent *P*-values (Mann-Whitney U test). (**b**) ROC curves for IgE, NSE and peripheral blood eosinophils. AUC values are shown for each parameter.

According to their allergic disease state, patients had significantly augmented levels of eosinophils and total IgE compared to HC, but there were no significant changes regarding disease severity (Table 1). IgE showed a positive correlation with eosinophil, monocyte and, to a lesser extent, lymphocyte blood count, underlying the relevance of these subsets in allergic asthma pathogenesis (Table 2). Since activation of eosinophils and macrophages has been associated to enhanced NSE levels under some pathological conditions^26,27^, we also undertook the measurement of this enzyme in serum samples. As Table 2 shows, it was found a positive correlation of IgE, eosinophils and monocytes with NSE (Table 2), which prompted us to examine the utility of this parameter as an additional marker of allergic asthma. As shown in Fig. 1a, NSE levels were augmented in AA compared to HC and tend to be around 14.6% higher in men than in women (Fig. 1a). Moreover, the area under the curve (AUC) of the ROC plot for NSE levels was close to AUC of total IgE and higher than AUC of blood eosinophils (absolute values) (Fig. 1b).

**Table 2:** Spearman correlation matrix of the study population.

VARIABLES	NSE	CRP	IgE	mCD14^#^	sCD14	Age
Leucocyte count	**0**.163*	**0**.221***	**0**.192***	−0.021	−0.035	**−0**.165***
Neutrophil count	0.003	**0**.190***	−0.027	−0.027	−0.034	−0.052
Lymphocyte count	0.077	**0**.107*	**0**.143***	−0.020	−0091	**−0**.152***
Monocyte count	**0**.303***	**0**.154***	**0**.336***	**0**.234***P	−0.021	**−0**.182***
Eosinophil count	**0**.279***	0.054	**0**.459***	**−0**.199***	−0.071	**−0**.273***
Basophil count	**0**.149***	−0.024	0.057	−0.034	−0.005	**−0**.113*
FEV1%	0.099 P	−0.088 P	−0.057 P	0.140 P	−0.177 P	**−0**.209***P
FEV1/FVC	0.106 P	−0.071 P	−0.051 P	0.151	−0.146 P	**−0**.375***P
TNF	−0.022	**0**.179***	−0.011	0.082	**0**.119**	**0**.198***
NSE		−0.004	**0**.322***	**−0**.215***	0.087	**−0**.128**
CRP			−0.033	−0.000	**0**.158***	**0**.119**
IgE				**−0**.264***	−0.054	**−0**.296***
mCD14^#^					−0.079	**0**.177***
sCD14						**0**.102*

CRP, C-reactive protein; IgE, immunoglobulin E; NSE, neuron specific enolase; P, patient population; TNF, tumour necrosis factor. *P < 0.05, **P < 0.01, ***P < 0.001. ^#^% of CD14^+^ monocytes.

No alterations were appreciated for C-reactive protein (CRP), IgG, IgA, IgM, or tumour necrosis factor (TNF) in AA. The influence of age was also taken into consideration, with a negative correlation with FEV1%, FEV1/FVC, IgE and leukocyte subtypes (mostly eosinophils), but a small positive association with TNF, CRP, mCD14 and sCD14 (Table 2).

### Allergic asthma enhances the number of peripheral blood monocytes, but causes a reduction of mCD14 in these cells

**Figure 2:**
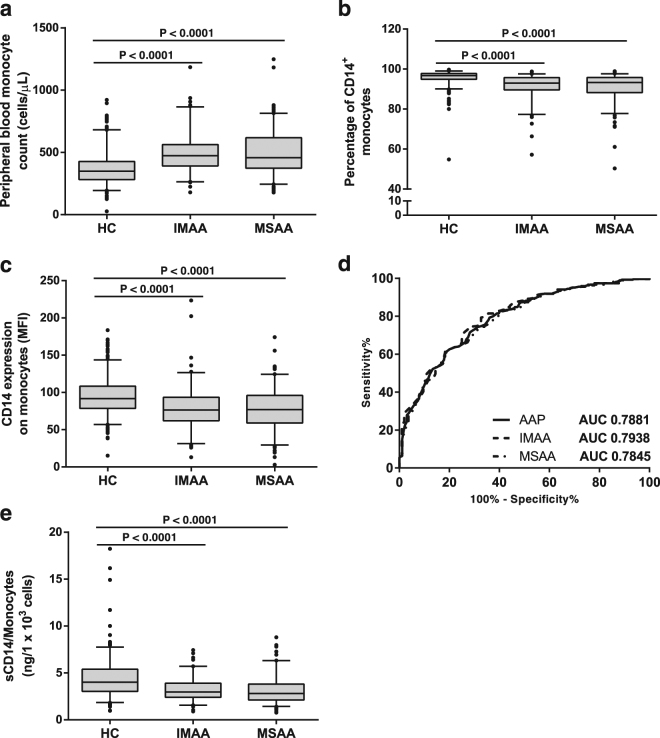
Monocyte count and mCD14/sCD14 levels in AA and HC. (**a**) Peripheral blood monocyte count in IMAA, MSAA and HC. Percentage of CD14^+^ cells (**b**) and mean fluorescence intensity (MFI) of CD14 on monocytes (**c**) in IMAA, MSAA and HC. (**d**) ROC curve for the percentage of CD14^+^ monocytes. (**e**) Relative sCD14 levels (ng/1 × 10^3^ monocytes) in serum samples from IMAA, MSAA and HC. Numbers on the graphs represent *P*-values (Mann-Whitney U test).

Complete blood count revealed an increased number of leukocytes in AA (HC 6.44 (5.39–7.75) × 10^3^ cells/μL vs. AA 6.93 (6.00–8.23) × 10^3^ cells/μL; *P* < 0.001). These differences were partially dependent on some innate subsets like eosinophils and monocytes, but not neutrophils or basophils (Table 1). Moreover, men had higher numbers of monocytes in peripheral blood than women (*P* < 0.0001), and asthma caused a slight but significant elevation of monocytes in both IMAA and MSAA (Fig. 2a). mCD14 was mainly expressed by monocytes (>90% CD14^+^) (Supplementary Figure 1), and asthma produced a decrease in the percentage of CD14^+^ monocytes (and mean fluorescence intensity/MFI values), without changes between IMAA and MSAA (Fig. 2b and c). Indeed, when a ROC curve was constructed for mCD14 values (%), the AUC gave a value of 0.7881 (whole asthmatics), which underlines the potential use of this parameter as an allergic asthma marker (Fig. 2d), even after segregating the patients in IMAA and MSAA.

Apart from monocytes, neutrophils and lymphocytes also contain CD14^+^ cells, but the expression of this marker is much lower than in monocytes (Supplementary Figure 1). Because of this, we assessed the differences between neutrophils and lymphocytes-associated mCD14 levels in AA and HC, but they were not influenced by the presence of allergic asthma (data not shown).

### Reduction of relative but not absolute sCD14 levels in allergic asthma

Despite the decreased levels of mCD14 in monocytes from AA, we did not reach significant differences in the serum concentration of sCD14 in IMAA, MSAA and HC (data not shown). Moreover, when a cut-off value of total IgE (80 IU/mL) was selected to maximize the true positive rate (70.76%) and minimize the false positive rate (15.88%) (Fig. 1b), sCD14 levels remained unaltered in donors with IgE < 80 IU/mL or ≥80 IU/mL.

As our results were supporting the presence of altered numbers of monocytes in asthmatic patients, and monocytes were the most likely source of sCD14, we evaluated the correlation between the absolute count of peripheral blood monocytes and the serum concentration of sCD14, with negative results (Table 2). In spite of this, sCD14 levels could still be influenced by the combined effects of increased monocyte count and decreased expression of mCD14 on this leukocyte subset (phenotype). Accordingly, sCD14 values were normalised with respect to the absolute number of circulating monocytes to maximize the effect of cell phenotype, finding in this case a significant reduction of sCD14 in AA compared to HC even after segregating by disease severity (Fig. 2e). We also detected higher levels of relative sCD14 in women compared to men (data not shown).

We next examined the mCD14-sCD14 correlation. In order to analyse the strength of this relationship, we have taken into account the absolute and normalized values of sCD14, as well as we have analysed the data as a whole or after segregating our results according to sex or donor group (healthy or diseased). However, we have found no connection between both parameters (Table 2, whole set of mCD14-sCD14 pairs), which underlines an independent regulation of both molecules. Apart from the monocyte count, alternative reasons might explain this low correlation, like other sCD14 sources (e.g. hepatocytes) or the existence of different genetic backgrounds. Regarding the first possibility, there were no differences in CRP or TNF between HC and AA (*P* > 0.05), or evidence of respiratory infection, even though there is very small correlation between CRP or TNF and sCD14 (Table 2). Therefore, we next analysed the influence of the *CD14* (−159 C/T) SNP genotype (rs2569190) on mCD14/sCD14 levels.

### Genotypic and allelic frequency distribution of *CD14* (−159 C/T) SNP in the study population

Genotype (TT, TC, CC) and allelic (C, T) frequencies were calculated in this study, and their distribution presented in Table 3. The whole population (χ^2^ = 0.014, *P* > 0.05), HC (χ^2^ = 0.002, *P* > 0.05), AA (χ^2^ = 0.007, *P* > 0.05), IMAA (χ^2^ = 0.041, *P* > 0.05), and MSAA (χ^2^ = 0.012, *P* > 0.05) were in HWE. Moreover, there was association between the (−159 C/T) SNP and the presence of asthma (Table 3), showing a decrease in the frequency of the T allele in AA (*P* = 0.016) and MSAA (*P* = 0.013), but not in IMAA compared to HC. Furthermore, the frequency of TT genotype of CD14 polymorphism were significantly lower in AA than in HC (*P* = 0.049), being only significant in MSAA (*P* = 0.039) when patients were segregated according to disease severity (Table 3).

**Table 3:** Genotype and allele frequencies of the *CD14* (−159 C/T) SNP in the study population.

	N	Genotype frequencies (%)				Allele frequencies (%)		
		TT	TC	CC		T	C	
HC	277	79 (28.5)	138 (49.8)	60 (21.7)		296 (53.4)	258 (46.6)	
AA	277	59 (21.3)	137 (49.5)	81 (29.2)	**χ**^**2**^ = **6**.**030** ***P*** = **0**.049*	255 (46.0)	299 (54.0)	**χ**^**2**^ = **5**.**776**^**a**^ ***P = *0**.016*
IMAA	108	26 (24.1)	52 (48.1)	30 (27.8)	χ^2^ = 1.851 *P* = 0.3963	104 (48.1)	112 (51.9)	χ^2^ = 1.531^a^ *P* = 0.2159
MSAA	169	33 (19.5)	85 (50.3)	51 (30.2)	**χ**^**2**^ = **6**.**444** ***P*** = **0**.039*	151 (44.7)	187 (55.3)	**χ**^**2**^ = **6**.**091**^**a**^ ***P = *0**.013*

AA, allergic asthmatics; HC, healthy controls; IMAA, intermittent-mild allergic asthmatics; MSAA, moderate-severe allergic asthmatics. ^a^Yates correction. *Significant difference.

As allelic and genotypic frequencies seem to be related to disease severity, we further divide MSAA into moderate (N = 129) and severe allergic asthmatics (N = 40). After this segregation, the association of the (−159 C/T) SNP was only maintained in severe asthmatics, both the allelic (T vs. C, χ^2^ = 6.478, *P* = 0.011) and genotypic (TT vs. CC, χ^2^ = 7.429, *P* = 0.024) frequencies, and it was lost in moderate asthmatics (T vs. C, χ^2^ = 2.749, *P* = 0.097; TT vs. CC, χ^2^ = 3.040, *P* = 0.219).

### Association between *CD14* (−159 C/T) SNP and allergic asthma risk

A summary of allergic asthma risk according to the different genetic models is presented in Table 4. We found an association between T allele and a decreased allergic asthma risk in the overall allergic asthma population (T vs. C: OR = 0.74, 95% CI = 0.59–0.94, *P* = 0.0162). Moreover, when AA were subset by disease severity (IMAA and MSAA vs. HC), this association was maintained only for MSAA (OR = 0.70, 95% CI = 0.54–0.92, *P* = 0.0136), while IMAA lost the allelic association (Table 4).

**Table 4:** Association between *CD14* (−159 C/T) SNP and allergic asthma risk.

		OR (95% CI)	χ^2^ (Yate’s correction)	*P*
AA (ALL)	TT + TC vs CC	0.67 (0.45–0.98)*	3.805	0.0511
	TT vs TC + CC	0.68 (0.46–1.00)	3.484	0.0620
	TT vs CC	0.55 (0.34–0.89)*	5.449	0.0196*
	TC vs CC	0.74 (0.49–1.11)	1.180	0.1704
	T vs C	0.74 (0.59–0.94)*	5.776	0.0162*
IMAA	TT + TC vs CC	0.72 (0.43–1.20)	1.300	0.2543
	TT vs TC + CC	0.79 (0.48–1.33)	0.566	0.4517
	TT vs CC	0.66 (0.35–1.23)	1.346	0.2460
	TC vs CC	0.75 (0.44–1.30)	0.781	0.3768
	T vs C	0.81 (0.59–1.11)	1.531	0.2159
MSAA	TT + TC vs CC	0.64 (0.42–0.99)*	3.630	0.0547
	TT vs TC + CC	0.61 (0.38–0.96)*	4.029	0.0442*
	TT vs CC	0.49 (0.28–0.85)*	5.767	0.0163*
	TC vs CC	0.72 (0.46–1.15)	1.572	0.2100
	T vs C	0.70 (0.54–0.92)*	6.091	0.0136*

AA, allergic asthmatics; CI, confidence interval; IMAA, intermittent-mild allergic asthmatics; MSAA, moderate-severe allergic asthmatics; OR, odds ratio; TC vs CC, heterozygote; TT vs CC, homozygote; TT vs TC + CC, recessive model; TT + TC vs CC, dominant model.

*Significant difference.

Regarding to the other genetic models, a significant association of *CD14* (−159 C/T) and allergic asthma risk was found between the homozygotes TT vs. CC (OR = 0.55, 95% CI = 0.34–0.89, *P = 0*.*0196*), and almost reached significance in a dominant model (TT + TC vs. CC: OR = 0.67, 95% CI = 0.45–0.98, *P = 0*.*0511*) (Table 4). Furthermore, after segregating by disease severity, it was found an association of allergic asthma risk with this SNP only in MSAA according to a recessive model (TT vs. TC + CC: OR = 0.61, 95% CI = 0.38–0.96, *P* = *0.0442*) or TT vs. CC genotype comparisons (OR = 0.49, 95% CI = 0.28–0.85, *P* = *0.0163*). Therefore, the results suggested that T allele and TT homozygote individuals have decreased risk of allergic asthma compared with C allele and CC homozygote carriers, respectively.

### The influence of the *CD14* (−159 C/T) SNP on CD14 levels

**Figure 3:**
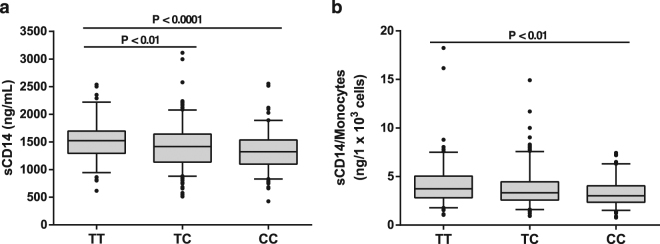
Impact of the *CD14* (−159 C/T) SNP (rs2569190) on sCD14 levels in serum. Absolute (ng/mL) (**a**) and relative (ng/1 × 10^3^ monocytes) (**b**) sCD14 levels in serum samples from TT, TC and CC donors (healthy and asthmatics). *Kruskal-Wallis One Way Analysis of Variance on Ranks, *P* < 0.001; Dunn’s Method was used for multiple comparisons (numbers on the graph represent *P*-values).

Although we have shown augmented peripheral blood monocyte count and decreased levels of mCD14 (Fig. 2a–c), the number of monocytes was not influenced by the *CD14* (−159 C/T) SNP genotype. We also failed to detect any change in mCD14 related to the SNP genotype (data not shown). In contrast (and regardless of whether they belong to the control group or to the asthmatic population), CC genotypes and to a lesser extent TC genotypes had lower concentrations of sCD14 (absolute values) than TT carriers, while no significant differences were observed between TC and CC subjects (Fig. 3a). This association was maintained between TT and CC carriers when sCD14 levels where normalised by the absolute count of monocytes (Fig. 3b). Therefore, this SNP could be partially responsible for the reduction of normalised sCD14 levels in AA and influence the severity of this disease (Fig. 2e).

## Discussion

In the current study, we report data supporting an increase of NSE and monocytes and a down-modulation of their mCD14 expression in allergic asthma regardless of disease severity. In addition, we detect a decrease of normalised sCD14 values in serum samples from asthmatics, suggesting the expansion of a CD14^low^ monocyte subset and the influence of the *CD14* (−159 C/T) SNP genotype. Indeed, we evidence an association of the T allele and TT genotype of *CD14* (−159 C/T) polymorphism with reduced risk of moderate-severe allergic asthma.

AA in our study have an atopic disease with eosinophilia, monocytosis and elevated levels of IgE and NSE. NSE is the neuronal isomer of the glycolytic enzyme 2-phospho-D-glycerate hydrolase, and a typical biomarker of small cell lung cancer^28^. Nevertheless, changes in non-malignant inflammatory lung diseases have also been found^27,29–32^, since this enzyme can translocate towards the cell surface upon proper stimulatory signals to enhance a proinflammatory response^33^. Our results support the presence of higher NSE levels in men, as previously reported by Collazos *et al*.^29^, but contrary to this work our asthma patient cohort displays above-normal NSE levels in serum. Monocytes/macrophages appear to be a possible source of NSE^27,33^, and increased numbers of monocytes as well as a correlation of them with NSE levels were demonstrated in our study. However, other plausible sources of NSE are eosinophils or injured epithelial cells during pulmonary infiltration^26^, while the neuronal distress or hypoxia occurring in the lung also could play a role during the disease^29^.

Apart from other cells as T and B lymphocytes, eosinophils, basophils or neutrophils, monocytes are gaining importance as regulators of inflammation in asthma and as key players in the pathogenesis^34–36^. Our results show the expansion of this subset in AA regardless the severity of the symptoms (i.e., IMAA and MSAA), as well as a reduction of mCD14, a monocyte marker associated to asthma^3^. In contrast, some authors have described no differences in the staining for mCD14^37,38^, even though these could be the result of a low statistical sample size. Reduced levels of mCD14 or TLR4 in AA makes biological sense^39^, as signal transduction through CD14/TLR4 leads to IL-12 secretion, a powerful inducer of TH_1_ polarization^13^. Therefore, attenuated mCD14 levels on antigen presenting cells (APCs) could favour TH_2_-driven allergic asthma^4,5,13,16^. This diminished number of mCD14 molecules on monocytes could arise as a result of several, and not mutually exclusive, mechanisms: (a) altered transcription/translation rates affecting protein abundance; (b) expansion of CD14^low^ monocyte subsets; (c) a vesicle- or enzymatic-mediated mechanism that release mCD14 from monocytes and should also affect sCD14 concentration.

The degree of mCD14 down-modulation on monocytes suggests the preferential expansion of a small CD14^low^ subset and not a globally altered transcription/translation rate. This, for example, is in line with the increased percentage of CD14^−/low^ monocytes upon *in vitro* culture in the presence of TSLP, an important cytokine in allergic asthma^40^. Monocytes are heterogeneous, with major (CD14^high^) and minor (CD14^low^) subsets^32^. CD14^high^ (“classical”) monocytes display a CD16/FcγRIII^-^ phenotype, while the less frequent CD16^+^ subset consists of both intermediate (CD14^high^CD16^+^) and non-classical (CD14^low^CD16^+^) subpopulations^33^. CD16^+^ monocytes, particularly the intermediate subset, are expanded in inflammation, severe asthma or upon allergen challenge^33,35^, in line with their pro-inflammatory nature^41^. A major constraint of our study is that we have not analysed CD16, but our results show a significant down-modulation of mCD14 in allergic asthma, which appears to rather support the expansion of CD14^low^CD16^+^ monocytes. These cells (non-classical subset) express high levels of CD80, CD86, and CD163, suggesting a high antigen presenting capability^42^. Furthermore, non-classical monocytes are in an advanced differentiation stage and they evidence high invading ability to infiltrate and differentiate into M2-type macrophages^43^, a subset related to allergic inflammation^44^.

CD14 can be released to medium from hepatocytes as an acute phase protein^45^. Although we saw a small correlation between sCD14 and CRP or TNF, the levels of these two last molecules had no changes between AA and HC, and our patients were in a steady-state of the disease. Excluding the hepatocyte origin, monocytes are the most likely cell source of sCD14. Down-modulation of mCD14 in monocytes from AA does not fit with either its shedding^2,6,7^ or the release of mCD14-enriched vesicles [http://exocarta.org/gene_summary?gene_id=12475] from these cells, because both processes should lead to a higher number of sCD14 molecules in the extracellular compartment, as happen during the acute phase^15,16,46,47^. However, patients in our study are in a chronic phase, where there is no relationship between monocyte count and sCD14 or mCD14-sCD14 correlation^16^. Therefore, our results only make sense considering a puzzling scenery with an elevation of monocyte numbers and enhanced frequencies of both CD14^high^ ^34^, but also CD14^low^ (our results) subsets of monocytes in asthma. Indeed, in our study only normalised serum levels of sCD14 were significantly reduced in patients. In agreement, sCD14 levels have been inversely correlated with IL-4-production^11^, total IgE^11^, or asthma severity^46^. However, some authors have detected higher levels^12^ or no differences^7,15^ of baseline sCD14 in peripheral blood from asthmatics. Hence, we cannot rule out the contribution of many potential confounding factors that explain these different results, like gene-gene or gene-environment interactions^11,15^.

One of the most studied CD14 polymorphisms in asthma is the *CD14* (−159 C/T) SNP (rs2569190)^11^. Previous studies investigating the association of this SNP with allergic asthma yielded variable results regarding to the strength and direction of association^3,17,24^. These contradictory results can be explained by differences in ethnicity, low sample size, age of patients or gene-environment interaction^21,25^. We performed our study in a well-defined population (Caucasian, adults, allergic asthmatics and mostly non-farmers), with a high sample size (277 AA vs. 277 HC), and two different disease severity grades (IMAA and MSAA). In agreement with others^11,17^, we show an association of the frequency of the C allele and the CC genotype with allergic asthma (whole asthmatics). More interesting, this association is also related to the disease severity, as it is only maintained in MSAA and, within this group, in severe asthmatics. Moreover, the risk of having moderate-severe allergic asthma (but not intermittent-mild asthma) is lower in carriers of the T allele (T vs. C) and TT genotype, following either a recessive model (TT vs. TC + CC) or after comparing TT vs. CC homozygotes. As other works have shown^5,11,12,18,19^, we evidence augmented sCD14 levels in subjects carrying the TT genotype but no association of this polymorphism with mCD14 levels on monocytes. This suggests an adverse role for the C allele, the CC genotype and the presence of low levels of sCD14/mCD14 in allergic asthma or atopy^11,46^, especially among adult and atopic subjects exposed to low levels of endotoxin, like our cohort^13^.

In summary, our findings show an increment in the serum levels of NSE, which could be used as a novel biomarker of allergic asthma. On the other hand, we also found a decrease in the expression of CD14 on monocytes from allergic asthmatic patients, probably related to an increase of CD14^low^ monocyte subset. Moreover, we evidence an association of the (−159 C/T) SNP in the CD14 promoter with allergic asthma, and a decreased risk of having moderate-severe allergic asthma in carriers of T allele and TT genotype. Furthermore, TT genotype is associated to higher levels of sCD14, pointing out a protective role for the T allele in this disease.

## Methods

### Subjects

The study population was recruited from January 2009 to December 2012, at the Unit of Pneumology and Allergy of the USC University Hospital Complex of Santiago de Compostela (CHUS) and the Pontevedra Hospital Complex (CHOP). This study population included 277 healthy controls (HC) and 277 allergic asthmatics (AA), consisting of 108 intermittent-mild allergic asthmatics (IMAA) and 169 moderate-severe allergic asthmatics (MSAA). Asthma and allergy diagnosis was confirmed according to Global Strategy for Asthma Management and Prevention criteria (GINA 2006, http://www.seicap.es/documentos/archivos/GINA2006general.pdf). All patients were in a stable phase for at least 4 weeks before the study initiation. Forced vital capacity (FVC), forced expiratory volume in 1 second (FEV1), and the FEV1/FVC ratio, were measured. Asthma diagnosis was confirmed by a positive broncodilatator test (>12% of FEV1 change after salbutamol) or metacholine challenge. Allergic sensitization was confirmed through a skin prick test or serum IgE specific to frequent allergens. Other variables were measured: smoking, pets at home, residence (rural/urban), profession, comorbidities, age of symptoms onset, asthma control, or number of visits to emergency units, family doctors or hospitals during the year prior to the study initiation. HC were selected from patients scheduled in the hospital for minor surgeries such as orthopedic surgery or inguinal hernia, and smoking and systemic diseases or allergies were used as exclusion criteria. The research was carried out according to The Code of Ethics of the World Medical Association (Declaration of Helsinki). The project was also approved by the Ethics Committee of Clinical Research of Galicia (2011/001), Spain, and all subjects signed informed consent statements.

### Flow cytometry assays

Venous peripheral blood was collected in ethylenediaminetetraacetic acid (EDTA) treated tubes (BD Vacutainer K2E). To analyse the expression of CD14 on peripheral blood leukocytes, CD14-FITC (Mouse IgG2a κ; BD Biosciences) or Isotype-FITC (Mouse IgG2a; BD Biosciences) were added to 100 μL of whole blood (30 min, room temp.). Then, red cells were lysed (BD FACS^TM^ Lysing Solution). Finally, 10 000 events were collected and analysed by means of a BD FACSCalibur flow cytometer. Data were examined using WinMDI 2.9 software (Joseph Trotter, La Jolla, CA. USA).

### Biochemical determinations

Biochemical determinations were carried out by an ADVIA^®^1650 analyser (SIEMENS Healthcare Diagnostics S.L., Berlin, Germany) while neuron-specific enolase (NSE) was measured with electrochemiluminescence analyser (MODULAR ANALYTICS Cobas E-601, Roche Diagnostics; Mannheim, Germany). The nucleated cell counting was performed using an ADVIA^®^2120 hematology counter (SIEMENS Healthcare Diagnostics S.L., Berlin, Germany).

Serum sCD14 levels were measured by means of an enzyme-linked immunosorbant assay (ELISA) with the Quantikine®Human sCD14 Immunoassay kit (R&D Systems, MN, USA). Optical densities were recorded at 450 nm and protein concentration was calculated from standard curves.

### Genomic DNA purification and *CD14* (−159 C/T) promoter SNP (rs2569190) studies

Genomic DNA was purified from whole blood (200 µL) with the QIAamp® DNA Mini Kit (QIAGEN, Melbourne, Australia). The subsequent study of the rs2569190 SNP in the CD14 promoter was conducted by the CEGEN-PRB2 USC node using the iPlex® Gold chemistry and MassARRAY platform, according to manufacturer’s instructions (Agena Bioscience, San Diego, CA). Genotyping assay (Polymerase chain reaction/PCR primers and single-base-extension/SBE primers) were designed using the Agena Bioscience MassARRAY Assay Designer 4.1 software. To avoid confusion in the mass spectrum, a tag (5-ACGTTGGATG-3) was added to the 5′ end of each PCR primer. Both SBE and PCR primer sequences are shown in Supplementary Table 1. PCR reaction was set up in a 5 µL volume and contained of template DNA (20 ng), 1 × PCR buffer, MgCl_2_ (2 mM), dNTPs (500 µM) and PCR enzyme (1 U/reaction). A pool of PCR primers was made at a final concentration of each primer of 100 nM (Metabion International AG, Germany). The thermal cycling conditions for the reaction consisted of an initial denaturation step at 95 °C for 2 minutes, followed by 45 cycles of 95 °C for 30 seconds, 56 °C for 30 seconds and 72 °C for 1 minute, and a final extension step of 72 °C for 5 minutes. PCR products were treated with shrimp alkaline phosphatase (1.5 U) by incubation at 37 °C for 40 min, followed by enzyme inactivation by heating at 85 °C for 5 min to neutralize unincorporated dNTPs.

The iPLEX GOLD reactions were set up in a final 9 µL volume and contained 0.222 × iPLEX buffer Plus, 0.222 × iPLEX termination mix and iPLEX enzyme (1.35 U/reaction). A SBE primer mix was made to give a final concentration of each primer between 0.52 µM and 1.57 µM (Metabion International AG, Germany). The thermal cycling conditions for the reaction included an initial denaturation step at 95 °C for 30 seconds, followed by 40 cycles of 95 °C for 5 seconds, with an internal 5 cycles loop at 52 °C for 5 seconds and 80 °C for 5 seconds, followed by a final extension step of 72 °C for 3 minutes. The next step was to desalt the iPLEX Gold reaction products with Clean Resin following the manufacturer’s protocol. The desalted products were dispensed onto a 384 Spectrochip II using an RS1000 Nanodispenser and spectra were acquired using the MA4 mass spectrometer, followed by manual inspection of spectra by trained personnel using MassARRAY Typer software, version 4.0. All assays were performed in 384-well plates, including negative controls and a trio of Coriell samples (Na10830, Na10831 and Na12147) for quality control. 10% random samples were tested in duplicate and the reproducibility was 100%.

### Statistics

Descriptive data are presented as either median (interquartile range; IQR1–3) or percentages. To assess the significance of changes between AA and HC, Mann–Whitney U two-tailed test or Kruskal–Wallis One Way Analysis of Variance on Ranks followed by Dunn’s multiple comparison test were used. Receiver Operating Characteristic (ROC) curves and Spearman’s association test were also employed, while differences in proportions were assayed by the χ^2^ test. To evaluate the association between *CD14* (−159 C/T) SNP and the risk of allergic asthma, Odd’s ratios (ORs) and 95% confidence intervals (CIs) were calculated according to different models: TT + TC vs. CC (dominant model), TT vs. TC + CC (recessive model), TT vs. CC, TC vs. CC, and T vs. C (allelic model). Hardy–Weinberg equilibrium (HWE) was calculated by using Pearson χ^2^ test. All analyses and graphs were conducted using GraphPad Prism version 6.0 (GraphPad Software, Inc., San Jose, California, USA). Data are presented in box and whisker plots, where median, 25 and 75 quartiles, 5–95 percentiles (error bars) and anomalous values are shown. The statistical signification was defined as *P* < 0.05.

## Electronic supplementary material

Supplementary Table 1 and supplementary Figure 1.
