# Supplementary material for: The CD14 (−159 C/T) SNP is associated with sCD14 levels and allergic asthma, but not with CD14 expression on monocytes
Source: Sci Rep. 2018 Mar 7;8:4147. doi: 10.1038/s41598-018-20483-1 (PMC5841440; doi:10.1038/s41598-018-20483-1)
Supplement: Supplementary file 1 — Supplementary Table 1 and supplementary Figure 1. [file 41598_2018_20483_MOESM1_ESM.pdf]

The CD14 (-159 C/T) SNP is associated with sCD14 levels and allergic asthma, but not with CD14 expression on monocytes.

Authors

Juan José Nieto-Fontarigo,[a] Francisco Javier Salgado,[a] María Esther San-José,[b] María Jesus Cruz,[c] Alejandro Casas-Fernández,[b] María-José Gómez-Conde,[b] Luis Valdés,[d] Miguel Ángel García-González,[e] Pilar Arias,[a] Montserrat Nogueira,[a] Francisco Javier González-Barcala.[f]

Affiliations

[a]Department of Biochemistry and Molecular Biology, Faculty of Biology-Biological Research Centre (CIBUS), Universidade de Santiago de Compostela, Santiago de Compostela, Spain.

[b]Clinical Analysis Service, University Hospital of Santiago de Compostela (CHUS), Santiago de Compostela, Spain.

[c]Department of Respiratory Medicine-Hospital Vall d'Hebron, Universitat Autònoma de Barcelona, Barcelona, Spain. Spanish Biomedical Research Networking Centre-CIBERES.

[d]Department of Medicine-University of Santiago de Compostela, Department of Respiratory Medicine-University Hospital of Santiago de Compostela, Health Research Institute of Santiago de Compostela (IDIS).

[e]Laboratory of Nephrology, Sanitary Research Institute (IDIS), Santiago de Compostela, Spain.

[f]Department of Medicine-University of Santiago de Compostela, Spanish Biomedical Research Networking Centre-CIBERES, Department of Respiratory Medicine-

University Hospital of Santiago de Compostela, Health Research Institute of Santiago de Compostela (IDIS).

Table of contents:

- Supplementary Table 1
- Supplementary Figure 1

Supplementary Table 1. PCR and SBE primers used for the iPLEX assay to assess the *CD14* (-159 C/T) polymorphism.

**Second PCR Primer***	acgttggatgAGACACAGAACCCTAGATGC
**First PCR Primer***	acgttggatgCAATGAAGGATGTTTCAGGG
**Amplicon length (bp)**	97
**UEP direction**	Reverse
**SBE unextended primer (UEP)**	AATCCTTCCTGTTACGG

*The lowercase letters in the PCR primer sequences are 5´-end tags.

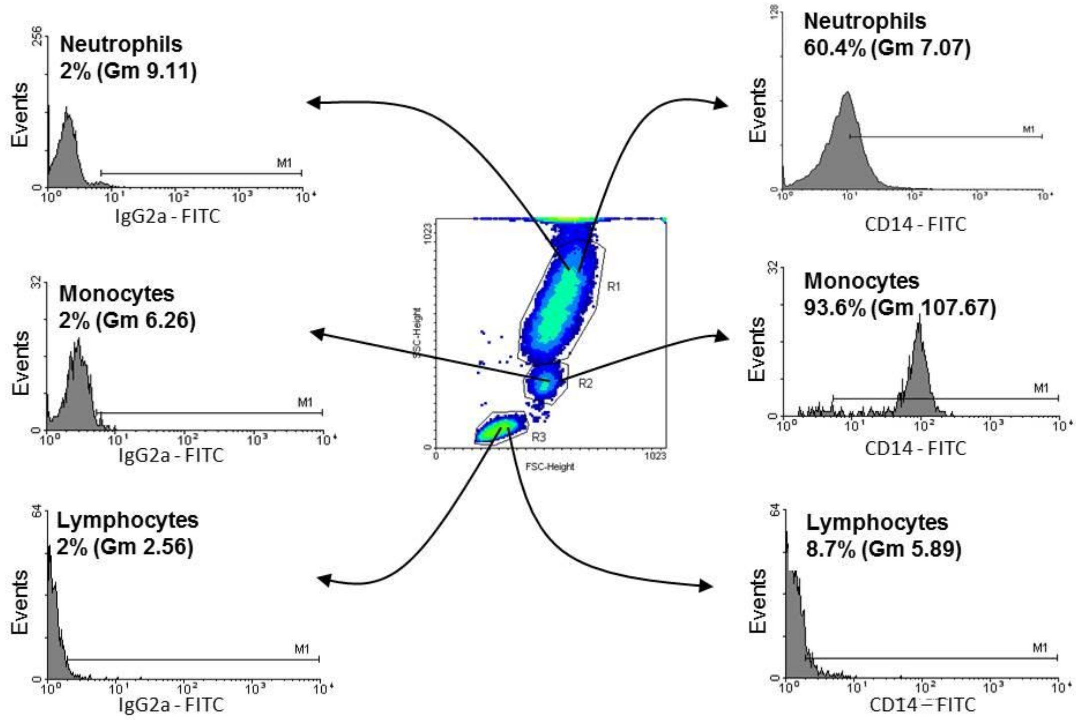

Supplementary Figure 1. CD14 expression in major peripheral blood leukocyte subpopulations. Leukocytes was marked with CD14-FITC or isotype antibody IgG2a κ-FITC. Data are shown as dot plots "forward scatter" (FSC) versus "side scatter" (SSC), where different leukocyte subpopulations are represented (neutrophils/R1, monocytes/R2 and lymphocytes/R3), and histograms, where mean fluorescence intensity (x-axis) is represented versus number of cells (y-axis) of every subpopulation (R1-R3). Left we can see the negative controls where 2% is choose to threshold value. A representative result is shown.
